# Seroprevalence and risk factors of *Toxoplasma* *gondii* infection in rabbit of local Algerian population

**DOI:** 10.14202/vetworld.2019.855-859

**Published:** 2019-06-20

**Authors:** Mina Henneb, Khaled Harhoura, Mohamed Amine Bekara, Safia Zenia, Miriem Aissi

**Affiliations:** 1 Department of Agronomy, Faculty of Sciences, University M’Hamed Bougara, Boumerdes, Algeria; 2 High National Veterinary School, Oued Smar, Algiers, Algeria; 3 Department of Biology, Hassiba Benbouali University, Chlef, Algeria

**Keywords:** Algeria, public health, rabbits, seroprevalence, *Toxoplasma gondii*

## Abstract

**Aim:**

The objective of this investigation was to determine the seroprevalence and identify the risk factors of *Toxoplasma gondii* infection in the rabbit of the local Algerian population from five districts of Northern Algeria.

**Materials and Methods:**

Blood samples of 350 rabbits were collected and analyzed for the presence of anti-*T. gondii* immunoglobulin G antibodies using the indirect enzyme-linked immunosorbent assay. Additional data concerning the farms and management practices were obtained through a questionnaire used in surveys and interviews.

**Results:**

The overall seroprevalence was 14.6% (51/350). The seroprevalence was significantly higher in outdoor rearing farms and was linked to the presence of animals from other species on the farm. A higher seroprevalence was found in older animals compared to younger ones. No difference in seroprevalence was noted with respect to the origin or gender of animals, type of cage, feed and water sources, presence of cats in the vicinity, hygiene status, or season.

**Conclusion:**

This study has shown that *T. gondii* prevalence in rabbits of the local population is relevant and may have important implications for public health in rural areas.

## Introduction

Most countries have one or more local rabbit breeds, which could play an important role in commercial production [1]. The local rabbit population is an important source of meat consumption in Algeria, particularly in rural areas [2]. This population is well adapted to reproduce in heat stress condition [3]. Several studies were undertaken to characterize the zootechnical performances of this population [3-5]. However, no epidemiological data are available about the presence of the different pathogens that can infect animals of this population. Health status is a key point for the development of meat rabbit production [6]. Toxoplasmosis is one of the most common worldwide diseases in the livestock industry (economic losses) and, being a zoonosis, represents a serious public health problem [7]. Rabbits are infected by ingesting food or water contaminated with *Toxoplasma gondii* oocysts from cat feces, or by the transmission of *T. gondii* to off-springs through transplacental infection [8,9].

In humans, multiple routes of transmission are possible. The consumption of raw or undercooked rabbit meat has been recognized as the main cause for humans to catch *T. gondii* infection [10-12]. Furthermore, handling of rabbit and other animals’ raw meat increases the risk of transmission of *T. gondii* to humans [13]. Rabbits and humans infected with toxoplasmosis do not usually show clinical symptoms; therefore, detection of antibodies is important in epizootiology [14]. Seroprevalence of *T. gondii* infection is related to geographical location, being higher in tropical countries and lower in colder countries [15]. In Algeria, many serological studies focusing on the detection of *T. gondii* antibodies in livestock were published. These studies showed a seroprevalence of 24% in sheep [16], 28% in horses and donkeys [17], and 14% and 7% in cattle and goats, respectively [18].

However, no studies have been published on rabbits in Algeria. The objective of the present study was to assess the seroprevalence of *T*. *gondii* and possible risk factors in farms raising rabbits from the local Algerian population.

## Materials and Methods

### Ethical approval and informed consent

This study was approved by the scientific council of High National Veterinary School, Algiers, Algeria. Informed consent was obtained from all the participants.

### Animals and study area

All rabbits used in this experiment belonged to the local population. The characteristics of this population were described by Zerrouki *et al*. [3]. The blood samples were collected from 350 rabbits raised in different farms (n=67) from five Northern Algeria districts. The sampling method used was stratified sampling, based on five strata, which were the five districts that represent the studied population. Within each stratum, we have carried out a random sampling: Boumerdes (18 farms/97 samples) (36°45’16’’N/3°26’34’’E), Tizi Ouzou (18 farms/96 samples) (36°42’42’’N/4°02’47’’E), Bouira (12 farms/60 samples) (36°22’29’’N/3°54’07’’E), Algiers (12 farms/60 samples) (36°45’08’’N/3°02’31’’E), and Blida (7 farms/37) (36°28’12’’N/2°39’49’’E) from September 2017 to June 2018. These regions belong to the same bioclimatic stage (subhumid) and located at an altitude ranging from 156 to 519m above sea level.

### Rabbits blood sampling

A blood sample (4 ml) was collected for each animal (n=350) during slaughtering in capped tubes without anticoagulant. Tubes were allowed to clot in a sloping position then centrifuged at 3.000 rpm for 10 min. Sera were collected and stored at −20°C until analysis.

### Study of risk factors

Epidemiological information regarding the district, type of housing, farm hygienic conditions and type of cages, age and gender of animals, type of feed and water source, and presence of cats and animals from other species was collected through a questionnaire that was administered face-to-face (the respondent was always the owner of the farm).

### Serodiagnosis

Enzyme-linked immunosorbent assay (ELISA) tests were performed to detect immunoglobulin G (IgG) antibodies against *T. gondii* in sera (Multi-species ID Screen^®^ Toxoplasmosis Indirect, IDVET, Montpellier, France) according to the manufacturers’ instructions. The cutoff for positive results was defined with an optical density (OD) of 0.350 (OD>0.350). Absorbance was measured at 450 nm with an automatic 96-well plate reader (Dialab EL×800). The results were expressed as sample/positive control (S/P) percentages according to the formula: S/P% = OD sample/OD positive control×100. Sera with S/P≤40% were deemed as negative, between 40% and 50% doubtful, between 50% and 200% positive, and ≥200% strong positive.

### Statistical analysis

Statistical data analysis was performed using the MASS of R software (R Development Core Team, 2016). Univariate and multivariate logistic regression models were applied to determine the association between toxoplasmosis and the selected risk factors. For univariate analysis, the relationships between categorical variables and toxoplasma seroprevalence were tested one by one using the Chi-square test (χ^2^). Variables with values of p<0.02 in univariate analysis at 95% confidence level were included into the multivariable logistic regression model. This model was fitted using a forward stepwise selection procedure of significant variables (p<0.05).

## Results

The presence of anti-*T. gondii* IgG antibodies was detected in 51 of the 350 test sera (14.6%, 95% confidence interval[CI]: 10.9-18.3%) (Table-1). The variables such as districts, presence of cats, gender, and season were not considered in the stepwise procedure of selection of multivariable logistic regression model (p>0.20). Only the effect of the house type, the presence of animals from species other than rabbits, and the age of the tested animals were included in the final model (p<0.05).

**Table-1:** Seroprevalence of *Toxoplasma gondii* in rabbits of local Algerian population and associated factors risk in different districts of Northern Algeria.

Variables	Categories	Total	Positive	Prevalence	Univariate		Multivariate	
	
					Crude OR (95% CI)	p-value	Adjusted OR (95% CI)	p-value
Districts	Algiers	60	7	11.7	Reference	0.47	-	-
	Blida	37	7	18.9	1.76 (0.05-0.27)		-	-
	Bouira	60	9	15	1.33 (0.46-3.99)		-	-
	Boumerdes	97	18	18.6	1.72 (0.69-4.69)		-	-
	Tizi Ouzou	96	10	10.4	0.88 (0.31-2.55)		-	-
House type	Modern rabbitry	290	27	9.3	Reference	<0.001	Reference	
	Artisan rabbitry	20	08	36.4	5.56 (2.06-14.26)		5.27 (1.55-17.55)	0.006
	Outdoor rearing	38	16	42.1	7.08 (3.30-15.11)		8.25 (3.22-21.80)	<0.001
Cages	Wire mesh	190	18	9.5	Reference	<0.001	-	-
	Craft cages	126	22	17.5	2.02 (1.04-3.98)		-	-
	Free range	34	11	32.4	4.57 (1.88-10.83)		-	-
Hygienic status	High	108	10	9.3	Reference	0.08	-	-
	Low	242	41	16.9	1.99 (0.96-4.16)		-	-
Feed	Concentrated	174	13	7.5	Reference	<0.001	-	-
	Mixed*	74	17	23	3.69 (1.69-8.22)		-	-
	Concentrated and grass	102	21	20.6	3.21 (1.54-6.89)		-	-
Water source	Pipe water	126	11	8.7	Reference	0.03	-	-
	Container	224	40	17.9	2.27 (1.15-4.81)		-	-
Presence of animals from other species	No	252	18	7.1	Reference	<0.001	Reference	<0.001
	Yes	98	33	33.7	6.60 (3.53-12.69)		6.58 (3.14-14.44)	
Cats in vicinity	No	11	3	27.3	Reference	0.20	-	-
	Yes	339	48	14.2	0.44 (0.12-2.06)		-	-
Season	Autumn	71	9	12.7	Reference	0.25	-	-
	Winter	117	13	11.1	0.86 (0.35-2.19)		-	-
	Spring	162	29	17.9	1.50 (0.69-3.54)		-	-
Age (month)	3-4.5	214	9	4.2	Reference	<0.001	Reference	
	5-7	64	16	25	7.59 (3.22-18.93)		7.43 (2.85-20.5)	<0.001
	≥8	72	26	36.1	12.87 (5.84-30.81)		11.42 (4.74-29.92)	<0.001
Gender	Male	151	18	11.9	Reference	0.28	-	-
	Female	199	13	16.6	1.46 (0.80-2.77)		-	-

* Mixed=Fruits, vegetables, grain, OR=Odd ratio, CI=Confidence interval

The multivariate model indicated that the house type had a significant impact on the seroprevalence of *T. gondii*. Indeed, using an outdoor rearing system (Odd ratio [OR]: 8.5, 95% CI: 3.2-21.8, p<0.001) or covered house (OR: 5.3, 95% CI: 1.5-17.5, p=0.006) significantly increased the risk of acquiring *T. gondii* infection compared to using a modern rabbitry. Furthermore, the presence of animals from other species in farms was found as a risk factor for *T. gondii* infection. There was an increased probability of infection when other animals were present in farms (OR: 6.6, 95% CI: 3.1-14.4, p<0.001) compared to their absence.

Moreover, the age of rabbits was identified as another risk factor. There was a higher risk of *T. gondii* infection in rabbits >8 months old (OR: 12.9, 95% CI: 5.8-30.8, p<0.001) and 5-7 months (OR: 7.6, 95% CI: 3.2-18.9, p<0.001) compared to those that were 3-4.5 months.

## Discussion

In Algeria, there are no data on *T. gondii* infection in rabbits. Recently, the rabbit population and its meat consumption have increased, and there is no regulation for the sale and slaughtering of these animals. Therefore, rabbits can contribute to increasing human toxoplasmosis, which was estimated previously at around 50% [19]. Determining toxoplasmosis seroprevalence in rabbits is a way to explore the potential risk of human infection caused by these animals. The main objective of this study was to investigate the seroprevalence and risk factors associated with *T. gondii* infection in rabbits of local Algerian population reared in different geographic locations. This investigation sampled five districts from Northern Algeria, characterized by a high number of breeders and meat rabbit consumption [20].

Different laboratory methods have been used by researchers to detect seroprevalence of *T. gondii* (modified agglutination test, ELISA, immunosorbent agglutination assay, indirect fluorescent antibody test and indirect hemagglutination assays, and Dye test) [21]. For this study, we chose to use an ELISA technique because it gives satisfying rapid, accurate, and sensitive results [22]. This technique was also used in cats and dogs [23], sheep [24] as well as some wild animals [25].

The results of our study showed that the overall seropositivity for *T. gondii* (14.6%) in rabbits was similar to the one found in Spain [26] and in Mexico [27]. However, the seroprevalence found in this study is lower compared to that reported in some Arabic and European countries (Iraq: 86%, [28], Slovakia: 74%, [29], and Poland: 22%, [30]), and slightly higher than that reported in Egypt (11%; [31]), in Czech Republic (10%, [32]), and China (10%, [33]). The variation in the prevalence among different parts of the world may be due to geographic and ecological factors, age, and husbandry practices of the animals [34], and differences in serological techniques, number of individuals, and sampling procedures [35,36]. However, in the current study, there were no strong differences in the climatic conditions of the five districts sampled. The potential risk factors that could be associated with seroprevalence of *T. gondii* infection were evaluated in the present study. In terms of rearing, higher seroprevalence was recorded in rabbits reared outdoor (42%), which is probably due to lower hygiene conditions, as per the findings previously reported by Wang *et al*. [33]. Moreover, in outdoor rearing systems, rabbits are often fed on grass that could be contaminated by *T. gondii* oocysts from cats which have higher resistance in the environment [37,38]. Another reason could be that rabbits reared outdoors would potentially come in contact with cats or other animals living in the area [39,40].

In our study, seroprevalence was significantly higher in farms where other animals were present (33.7% vs. 7.1%). It was reported that the presence of animals from different species such as goats, sheep, or cattle in areas where rabbits are raised increases the chance of *T. gondii* infection mainly caused by feeding on grass, which can be contaminated by oocysts [38]. In addition to that, *T. gondii* infection may be transmitted to carnivorous mammals by ingesting infected prey such as birds and rodents [41].

The relationship between age and rabbit toxoplasmosis showed that the prevalence was higher in rabbits older than 8 months of age. This is in agreement with several previous studies [6,14]. Similar results were reported on sheep, goats and camels [42], cats [20], and dogs [43]. This may be related to the fact that rabbits that lived longer might be more likely to be exposed to infections from different sources [12]. The higher seroprevalence in older rabbits suggests that these infections are mainly maintained by horizontal rather than vertical transmission. However, Alvarado-Esquivel *et al*. [27] and Uhliková and Hübner [8] have reported a higher *T. gondii* seroprevalence in young rabbits (age category: 0.3-1 month) and they also discussed the possibility of transplacental transmission. In our study, all rabbits used were older than 3 months.

## Conclusion

The results of this first work have not only shown that *T. gondii* infection is widespread among rabbits of the local population in rural areas of Northern of Algeria but also provide information about the different risk factors contributing to its transmission. Despite the lower seroprevalence found in this study, it is possible that the infection, or toxoplasmosis, may also be prevalent in human beings living in the studied rural areas transmitted by rabbit meat consumption. Therefore, further studies, including other regions where rabbits are frequently consumed, should be conducted to pinpoint the prevalence in humans and rabbits. Finally, considering zoonotic potential and public health concerns, awareness of farmers in rural areas on means of transmission and prevention of *T. gondii* infection should be raised through education.

## Authors’ Contributions

MH and KH designed all steps of the study. MAB and SZ analyzed the data, MA reviewed the manuscript, and MH wrote the manuscript draft and collected all data. All authors read and approved the final manuscript.

## References

[1] Szendrő K, Szendrő Z.S, Matics Z.S, Zotte A.D, Odermatt M, Radnai I, Gerencsér Z.S (2015). Effect of genotype, housing system and hay supplementation on performance and ear lesions of growing rabbits. Livest. Sci.

[2] Berchiche M, Kadi S.A (2002). The Kabyle rabbits (Algeria). In:Rabbit Genetic Resources in Mediterranean Countries.

[3] Zerrouki N, Bolet G, Berchiche M, Lebas F (2005). Evaluation of breeding performance of local Algerian rabbit population raised in the Tizi-Ouzou area (Kabylia). World Rabbit Sci.

[4] Berchiche M, Kadi S.A, Lebas F (2000). Valorization of wheat by-products by growing rabbits of local Algerian population. World Rabbit Sci.

[5] Belhadi S (2004). Characterization of Local Rabbit Performance. Proceeding of 8^th^World Rabbit Congress, Puebla (Mexico).

[6] Meng Q.F, Wang W.L, Ni X.T, Li H.B, Yao G.Z, Sun X.L, Wang W.L, Cong W (2015). Seroprevalence of *Encephalitozoon cuniculi* and *Toxoplasma gondii* in domestic rabbits (*Oryctolagus cuniculus*) in China. Korean J. Parasitol.

[7] Dubey J.P (2010). Toxoplasmosis of Animals and Humans.

[8] Uhliková M, Hübner J (1973). Congenital transmission of toxoplasmosis in domestic rabbits. Folia Parasitol.

[9] Remington J.S, Thulliez P, Montoya J.G (2004). Recent developments for diagnosis of toxoplasmosis. J. Clin. Microbiol.

[10] Webster J.P (2010). Review of toxoplasmosis of animals and humans. Parasit. Vectors.

[11] Alvarado-Esquivel C, Torres-Berumen J.L, Estrada-Martínez S, Liesenfeld O, Mercado Suarez M.F (2011). *Toxoplasma gondii* infection and liver disease:A case-control study in a Northern Mexican population. Parasit. Vectors.

[12] Machacova T, Bartova E, Sedlak K, Budikova M, Piccirillo A (2015). Risk factors involved in transmission of *Toxoplasma gondii* and *Neospora caninum* infection in rabbit farms in Northern Italy. Ann. Agric. Environ. Med.

[13] Almerıa S, Calvete C, Pagés A, Gauss C, Dubey J (2004). Factors affecting the seroprevalence of *Toxoplasma gondii* infection in wild rabbits (*Oryctolagus cuniculus*) from Spain. VetParasitol.

[14] Abou Elez R.M.M, Hassanen E.A.A, Tolba H.M.N, Elsohaby T.I (2017). Seroprevalence and risk factors associated with *Toxoplasma gondii* infection in domestic rabbits and humans. Vet. Parasitol.

[15] Robert-Gangneux F, Darde M.L (2012). Epidemiology of and diagnostic strategies for toxoplasmosis. ClinMicrobiolRev.

[16] Dechicha A.S, Bachi F, Gharbi I, Gourbdji E, Ammi D.B, Brahim-Errahmani M, Guetarni D (2015). Sero-epidemiological survey on toxoplasmosis in cattle, sheep and goats in Algeria. Afr. J. Agric. Res.

[17] Mohamed-Cherif A, Ait-Oudhia K, Khelef D (2015). Detection of anti-*Toxoplasma gondii* antibodies among horses (*Equus caballus*) and donkeys (*Equus asinus*) in Tiaret province, Northwestern Algeria. Rev. Méd. Vét.

[18] Ait-Oudhia K, Mohamed-Cherif A (2015). Sero-epidemiological survey of toxoplasmosis in cattle, sheep and goats in Algeria. J. Bacteriol. Parasitol.

[19] Messerer L, Bouzbid S, Gourbdji E, Mansouri R, Bachi F (2014). Séroprévalence de la toxoplasmose chez les femmes enceintes dans la wilaya d'Annaba, Algérie. Rev. Epidemiol. Sante Publique.

[20] Saidj D, Aliouat S, Arabi F, Kirouani S, Merzem K, Merzoud S, Merzoud I, Ainbaziz H (2013). La cuniculture fermière en Algérie:Une source de viande non négligeable pour les familles rurales. Livest. Res. Rural Dev.

[21] Suyog S, Bishwas S, Subir S, Yugal R.B (2018). Seroprevalence of *Toxoplasma gondii* in sheep in different geographical regions of Nepal. Vet. Anim. Sci.

[22] Iovu A, Györke A, Mircean V, Gavrea R, Cozma V (2012). Seroprevalence of *Toxoplasma gondii* and *Neospora caninum* in dairy goats from Romania. VetParasitol.

[23] Scarpulla M (2009). Detection of anti-*Toxoplasma gondii* Antibodies in Dogs and Cats:Comparison of Indirect Immunofluorescence, Direct Agglutination, and Indirect ELISA. In:Proceeding WAVLD, Madrid (Spain).

[24] Mangili P.M (2009). Development and Evaluation of the Performance of an in House ELISA to be used for the Indirect Diagnosis of Toxoplasmosis in Sheep. In:Proceeding of SIDILV, Parma (Italy).

[25] Gheorghe D, Mihăiţă A, Rareş T.O, Marius S, Ilie A.B, Ionela H (2011). Epidemiological remarks on *Toxoplasma gondii* infection in Timişoara zoo. Sci. Parasitol.

[26] Almerıa S, Calvete C.B, Pagés A, Gauss C, Dubey J.P (2004). Factors affecting the seroprevalence of *Toxoplasma gondii* infection in wild rabbits (*Oryctolagus cuniculus)* from Spain. Vet. Parasitol.

[27] Alvarado-Esquivel C, Alvarado-Esquivel D, Villena I, Dubey J.P (2013). Seroprevalence of *Toxoplasma gondii* infection in domestic rabbits in Durango state, Mexico. PrevVetMed.

[28] Aghwan S.S, Al-Taee A.F, Suliman E.G (2010). Detection of *Toxoplasma gondii* infection in domestic rabbits by using multiple techniques. Iraqi J. Vet. Sci.

[29] Luptakova L, Balent P, Valencakova A, Novotny F, Petrovova E (2009). Serological detection of antibodies to *Toxoplasma gondii* in animals kept in households. Folia Vet.

[30] Sroka J, Zwolinski J, Dutkiewicz J, Tos-Luty S, Latuszynska J (2003). Toxoplasmosis in rabbits confirmed by strain isolation:A potential risk of infection among agricultural workers. Ann. Agric. Environ. Med.

[31] Ashmawy K.I, Abuakkada S.S, Awad A.M (2011). Seroprevalence of antibodies to *Encephalitozoon cuniculi* and *Toxoplasma gondii* in farmed domestic rabbits in Egypt. Zoonoses Public Health.

[32] Neumayerova H, Jurankova J, Jeklova E, Kudlackova A, Faldyna M, Kovarcik K, Janova E, Koudela B (2014). Seroprevalence of *Toxoplasma gondii* and *Encephalitozoon cuniculi* in rabbits from different farming systems. Vet. Parasitol.

[33] Wang S, Yao Z, Li L, Pan Y, Li P, Nan X, Xie Q, Zhang Z (2018). Seroprevalence of *Toxoplasma gondii* and *Encephalitozoon cuniculi* among domestic rabbits in central China. Parasite.

[34] Tasawar Z, Aziz F, Lashari M.H, Shafi S, Ahmad M, Lal V, Hayat C.S (2012). Seroprevalence of human toxoplasmosis in southern Punjab, Pakistan. Pak. J. Life Soc. Sci.

[35] Ramzan M, Akhtar M, Muhammad F (2009). Seroprevalence of *Toxoplasma gondii* in sheep and goats in Rahim Yar Khan (Punjab), Pakistan. Trop. Anim. Health Prod.

[36] Zewdu E, Agonafir A, Tessema T.S, Tilahun G, Medhin G, Vitale M, Marco V.E, Cox D, Vercruysse J, Dorny P (2013). Seroepidemiological study of caprine toxoplasmosis in east and West Shewa zones, Oromia regional state, central Ethiopia. ResVetSci.

[37] Figueroa-Castillo J.A, Duarte-Rosas V, Juárez-Acevedo M, Luna-Pastén H, Correa D (2006). Prevalence of *Toxoplasma gondii* antibodies in rabbits (*Oryctolagus cuniculus*) from Mexico. J. Parasitol.

[38] Shin H.G, Lee S.E, Hong S.H, Kim S.M, Choi Y.K, Park H.J, Seo K.W, Song K.H (2013). Prevalence of *Toxoplasma gondii* infection in rabbits of Korea by serological tests and Nested Polymerase Chain Reaction. J. Vet. Med. Sci.

[39] Dubey J.P (2004). Toxoplasmosis, a waterborne zoonosis. VetParasitol.

[40] Yekkour F, Aubertc D, Mercierd A, Muratd J.B, Khamesa M, Nguewaf P, Ait-Oudhia K, Villenac I, Boucheneg Z (2017). First genetic characterization of *Toxoplasma gondii* in stray cats from Algeria. Vet. Parasitol.

[41] Ahmad N, Qayyum M (2014). Seroprevalence and risk factors for toxoplasmosis in large ruminants in Northern Punjab, Pakistan. J. Infect. Dev. Ctries.

[42] Tilahun B, Hailu Y.T, Tilahun G, Ashenafi H, Shimelis S (2018). Seroprevalence and risk factors of *Toxoplasma gondii* infection among domestic ruminants in East Hararghe zone of Oromia Region, Ethiopia. Vet. Med. Int.

[43] Wu S.M, Huang S.Y, Fu B.Q, Liu G.Y, Chen J.X, Chen M.X, Yuan Z.G, Zhou D.H, Weng Y.B, Zhu X.Q, Ye D.H (2011). Seroprevalence of *Toxoplasma gondii* infection in pet dogs in Lanzhou, Northwest China. Parasite Vectors.

